# Navigating choice in multiple sclerosis management

**DOI:** 10.1186/s42466-019-0005-5

**Published:** 2019-02-28

**Authors:** Ralf A. Linker, Andrew Chan

**Affiliations:** 1 Department of Neurology, University of Regensburg, Universitätsstr. 84, 93053 Regensburg, Germany; 2 Ambulantes Neurozentrum, Inselspital, Bern University Hospital, Freiburgstr. 4, 3010 Bern, Switzerland

**Keywords:** MS therapy, Immunomodulation, Personalized medicine, Treatment monitoring, Disease management

## Abstract

**Background:**

With the advent of modern immunotherapies for relapsing-remitting multiple sclerosis (RRMS) and the increasing amount of treatment options on the market, MS has evolved as a treatable disease. Yet, at the same time, new challenges for the treating neurologists arise.

**Main body:**

This review article covers some of these challenges, including when and how to start treatment, treatment monitoring, and finally considerations on what the increasing choice in treatment options brings to disease management and longer-term planning. Among others, these important issues comprise pregnancy, treatment sequencing, switching or even stopping treatment.

**Conclusion:**

The ultimate goal for navigating choices in RRMS management is to choose the right drug for the right patient at the right time Throughout the article, there is a strong focus on practical aspects and individual decision making in MS to meet the concept of personalized medicine.

## Background

In the past 5 years, more than five new treatment options entered the stage for immunotherapy of relapsing-remitting multiple sclerosis (RRMS), expanding the treatment armamentarium to more than a dozen substances in total [9]. Some of these compounds represent reformulations of already existing treatment options while others make use of bona fide new treatment concepts [11, 33]. While there is still no cure for MS yet and many unmet needs like optimal treatment of chronic progressive disease stages abound, RRMS has evolved as a treatable condition. In fact, considerable evidence points at long-term effects of modern immunotherapies on disability and also quality of life in cohorts of mostly younger RRMS patients (e.g. for beta interferons, [22]).

Yet, with the advent of modern RRMS immunotherapy, new challenges for the treating neurologists arise. In this review, we will cover some of these new aspects including when and how to start treatment, treatment monitoring, and finally considerations on what the increasing choice in treatment options brings to disease management and longer-term planning including important issues like pregnancy, treatment sequencing, switching or even stopping treatment. Throughout the article, there will be a strong focus on practical aspects and individual decision making to meet the concept of personalized medicine [19].

### How to deal with increasing numbers of disease modifying therapies?

When thinking about treatment choices for RRMS, it is appropriate to discuss if the ever-expanding range of new disease modifying therapies (DMT) is really useful or if it is just making treatment regimen too complex while bringing only marginal benefits to patients. In fact, accommodating the range of new treatments may be challenging to the general neurologist managing MS patients, and in many instances a few substances may suffice to cover uncomplicated MS phenotypes. To date, MS is still a chronic disease with a significant risk of permanent disability impacting on the social roles in everyday life for the mostly young patients [46]. In addition, there is considerable heterogeneity regarding disease mechanisms as well as regarding individual patient profiles and needs [34]. Thus, at first glance, an appropriate range of treatment choices seems clearly beneficial.

Yet, it may be worthwhile to take a look at the evolution of immunomodulatory MS therapy over the past 20 years. With only interferon beta preparations and later also glatiramer acetate as injectable immunomodulators on the one hand and some limited data for time-honoured immunosuppressants like azathioprine, cyclophosphamide or mitoxantrone on the other, treatment choices were limited, but also easier to choose from. With more options at hand over the last years, there certainly is greater complexity and responsibility for patients as well as treating physicians alike, especially with respect to treatment adherence and monitoring.

Since the advent of natalizumab as the first monoclonal antibody for MS treatment in 2007, there is an ongoing process of learning about the benefits and risks of more than a dozen therapies. In particular, important points are what to screen for at baseline and what to tell patients before starting them on a new therapy. Different monitoring protocols have been instituted to manage the distinct risk profiles of single compounds with the rare but imminent risk for progressive multifocal encephalopathy (PML) on treatment with natalizumab as a very prominent example [17].

However, there has been much inspiration and hope that the newer compounds may harbour the potential to yield a higher efficacy for the individual MS patient. The pivotal AFFIRM trial on natalizumab started enrolling patients more than 15 years ago thus marking the start of an era of new therapeutic options [41]. In this trial, natalizumab reduced the rate of clinical relapse at 1 year by 68%, the risk of sustained progression of disability over 2 years by 42%, and led to an 83% reduction in the accumulation of new or enlarging hyperintense T2 lesions, as detected by cranial magnetic resonance imaging (MRI). These results paved the way for the concept of freedom of disease activity [15] and later no evidence of disease activity (NEDA, [2]) as new compound trial read-out which may also impact on our treatment goals in everyday practice [13]. Over the last 10 years, we now additionally gained clinical experience with the newer DMTs and new concepts arose, like selective or pulsed immune reconstitution therapy (PIRT) with alemtuzumab [60] and cladribine [59] as new players in the armamentarium of immunotherapy and bone marrow transplantation as an ultimate therapy concept. PIRT may offer lasting effects without continuous treatment for a significant proportion of patients. However, at this time point, it is not entirely clear if the proposed immune reconstitution is an immunologically valid scaffold for the observed sustained clinical effects.

Furthermore, data from the phase II and III randomized clinical trials were increasingly supplemented by data from extension studies and real-world evidence from registries which are of help to obtain some view on comparative efficacy of compounds as well as on more rare side effects [57]. A good example is the MS Base registry with large patient numbers allowing for propensity matched scoring analyses. In a recently published study on RR-MS patients from this registry, Kalincik and co-workers compared 189 patients given alemtuzumab, 2155 patients given interferon beta, 828 patients given fingolimod, and 1160 patients given natalizumab [20]. They found similar effects of alemtuzumab and natalizumab on annualized relapse rates. While alemtuzumab seemed superior to fingolimod and interferon beta in mitigating relapse activity, natalizumab seemed superior to alemtuzumab in enabling recovery from disability. While such studies may be helpful in informing on some efficacy measures between distinct compounds, treatment decisions between e.g. alemtuzumab and natalizumab may be primarily governed by further factors like treatment concept (see above) or side effects profile.

Despite all positive data with new compounds, there is still an ongoing debate on the magnitude of effects which can be achieved with our treatment armamentarium. Here, data from a large Italian MS cohort may be of interest, in which approximately 70% of patients have been on treatment since 1995 [5]. For the first three epochs of diagnosis, 1980–1990, 1991–1995 and 1996–2000, there was a similar rate of patients reaching a milestone EDSS of 6.0 (i.e. walking with a cane). For patients diagnosed during the epoch from 2001 to 2005, this rate was reduced by 37%, and for those diagnosed in 2006–2010, this rate was reduced by 46%. While it may be too early to draw conclusions for the cohort diagnosed since 2011, these data suggest that the course of MS is significantly influenced by the epoch of diagnosis with new treatment options probably being an important variable. Thus, new treatment options may bring significant value to the clinical outcomes for patients, and therefore their quality of life.

Yet, with a large array of options, the responsibility to make the right choice for the individual patient is also increasing. At this point, it may be worthwhile to turn to guidelines and how they can inform about decision making. A good example may be the German guidelines for immunotherapy of RRMS (www.kompetenznetz-multiplesklerose.de) which evolved from the escalation concept of the German MS Consensus Group [38]. In this guideline, treatments are grouped, or rather lumped together, for treatment of mild and moderate versus active and highly active disease courses. In some cases, the position of a single compound is rather based on perceived risk profiles and recommendations of the summary of product characteristics than pure efficacy. Furthermore, it is important to note that this guideline deliberately did not specify concrete criteria to define “active” or “highly active” and only sets a scope to leave room for individual treatment decisions. Finally, the guideline dates from 2014. An update is currently under revision and European guidelines with even more general recommendations were recently published [36, 37]. The limitation of such guidelines can also be witnessed in the case of the recent newcomer daclizumab, which has been available in Germany since 2016 and has not been mentioned in the guidelines. While the initial European label allowed its use in all relapsing MS patients, fatal cases of liver failure first led to restrictions in its use. After fatal cases of drug reaction with eosinophilia and systemic symptoms and severe inflammatory brain disorders, e.g. anti-NMDA-encephalitis and anti-GFAP-IgG associated encephalitis, daclizumab was finally withdrawn from the market [8, 32, 43, 47].

Thus, guidelines may only set a framework but are of limited help to inform individual decision making for single patients.

In a practical approach, three main domains for guiding treatment decisions may be considered: disease activity and prognosis, potential drug issues and the individual patient profile. Regarding disease activity and prognosis, the distinction between active and inactive disease is worthwhile to consider as put forward in the Lublin classification of the clinical course of MS in 2013 [31]. In addition, patients with rapidly evolving, highly active disease may be viewed separately. While there is no consensus on a definition of this group, the EMA recently proposed two relapses in the last year regardless of therapy or one relapse under therapy with MRI evidence of active disease (contrast enhancement and at least 9 T2 lesions) as criteria relevant for the label of cladribine (http://www.ema.europa.eu/docs/en_GB/document_library/EPAR_-_Product_Information/human/004230/WC500234561.pdf). Regarding drug related issues, one may consider tolerability, the individual safety profile, monitoring frequency, and drug effects. Tolerability issues may classically comprise flu-like symptoms or also gastrointestinal side-effects. When discussing drug safety, autoimmunity, risks for neoplasms and the risk for infections, particularly opportunistic infections (e.g. PML), are of paramount interest. Individual pharmacodynamics and pharmacokinetic properties of single compounds play an important role for drug-drug interactions, carry-over infections, rebound effects and sequencing of drugs. Regarding patient profiles, there is an important role of adherence, co-morbidities (skin, heart, liver, renal function, other autoimmune diseases, hematopoietic abnormalities, [26]) and, finally, personal factors. These factors are nowadays of particular importance and comprise personal views on pregnancy and family planning, work, travel, spare-time activities, daily structure and others, which in turn may influence quality of life and treatment choices [30].

In summary, it is important to be aware of all available treatment options for MS. With the unique characteristics of every patient and the increasing number of individual factors on disease activity, drug properties and patient profiles to be considered, a great range of therapeutic options is highly welcome [19]. Hypothetically, heterogeneity of pathomechanisms can be met by different modes of action of the broad range of immunotherapies like recently proposed for plasma exchange which may be more effective in histopathological patterns I and II [54].

### Monitoring MS disease activity

Disease monitoring under immunotherapy is in the focus of an at times controversial debate within the MS community. Prevailing questions are: What should we measure? How should we measure? Is there a threshold of acceptable disease activity, or should we be switching therapy at the first sign of any uncontrolled activity? To further set the scene for this discussion, it may be worthwhile to take a look at disease monitoring in the setting of phase III clinical MS trials, and then compare it to the approach in clinical practice. In a clinical phase III trial, patients are routinely seen every 4 weeks to 3 months. Typical outcomes include assessment of relapse rates, disability as measured by multiple sclerosis functional composite and EDSS every 3 months by a certified rater, and additionally cranial MRI, as evidenced in the NEDA concept (see above). Yet, in the future, patients reported outcomes (PRO, e.g. MSIS-29), brain atrophy, or cognitive measures like the symbol digit modalities test (SDMT) every 6 months may add to this concept [12]. Along this line, visual quality of life is a potentially under-recognized parameter, which can be quantified using the NEI-VFQ 25 [16]. In addition, efficacy monitoring in clinical trials is more frequent than expected in clinical practice, and nowadays engages a range of techniques aiming at maximising objectivity (e.g. low contrast visual acuity charts, optical coherence tomography; OCT etc.). For OCT, feasibility of automatic segmentation with manual correction in specific macular areas was shown even in a multi-center setting [39]. In real-life outpatient practice, there is considerably less time (sometimes only a few minutes), and far fewer resources to assess the patient for ongoing disease activity. While access to MRI may be good in many health care settings nowadays, the quality of the radiologist and the report may vary. In addition, clinical studies require a quantitative MRI report. Yet, real world MRI reports are often very descriptive without stating clear quantitative results relative to the previous scan.

When discussing what to measure, it is important to understand current concepts of MS disease evolution. MS is often described by the iceberg analogy with the clinical manifestations above the water representing a small fraction of the full disease pathology [14]. In fact, MS is a microscopic disease and clinical endpoints only represent the literal tip of this iceberg. In accordance, focal MRI only represents the tip of sub-clinical disease processes which include grey matter or changes of the normal appearing white matter (NAWM). It has been well described that MRI may pick up damage before clinically relevant changes are observed. There is very convincing data that new T2-lesions detected by MRI are a good predictor of later relapses. A range of brain volumetric changes may very well indicate the level of end organ (CNS) damage and predict later disability. However, lack of standardization, among others owing to high technical variability, may complicate implementation in clinical routine [3]. Beyond MRI techniques, modern concepts are trying to validate more sensitive, simpler measures of CNS damage and focus on cognition (see below), optical coherence tomography [40] or also serum levels of the neurofilament light chain with the very sensitive single molecule array (SIMOA) technique [23, 28].

Yet, nowadays, cranial MRI is most widely employed to sensitively assess adequate disease control under immunotherapy. In addition to many scoring systems for disease activity, the Rio Score and the modified Rio Score represent two widely discussed approaches that may define adequate versus inadequate disease control under beta interferon therapy. While the “classical” Rio Score combines MRI measures, relapses and EDSS [44], the modified Rio score removes the requirement to measure the EDSS objectively at every visit [49]. Sormani and co-workers applied the modified Rio score to phase 3 study data with interferon beta 1a three times weekly. They further refined its use by adding a 6-month follow up MRI for those patients with a score of 1 at year one [50].

Thus, they were able to classify patients as either “interferon responders” or “non-responders” with the “non-responder” group being just as likely to progress as an untreated group. In addition, the modified Rio score may also identify responders to oral therapies like fingolimod or teriflunomide [52] pointing at a general and not an interferon beta therapy specific principle. While it is not entirely clear if the same criteria could be used to stratify responders and non-responders to any other treatment, such scoring systems provide some guidance what thresholds of MRI activity may not be tolerated if the EDSS is not included as part of a scoring system*.* In addition, volumetric measures may also be helpful as shown in a meta-analysis of 13 placebo-controlled RRMS trials involving more than 18.000 MS patients. This study revealed that the effects of a given therapy on T2 lesion volume and the rate of whole brain volume change combined may explain 75% of its impact on disability progression [51].

For a practical approach, one may refer to the MAGNIMS guidelines for MR imaging in MS [58]. It is recommended to perform the first monitoring scan some 6–12 months after treatment initiation (“rebaselining”) and then yearly thereafter. In order to co-register images, patients need to be scanned in the same scanner with the same protocol including field strength of ≥1.5 T with a maximum slice thickness of 3 mm (or, better, 3D scans) and identical sequence parameters, orientation and slice positioning. For detecting new or enlarging lesions proton density and/or T2-FLAIR and T2 weighted fast or turbo spin-echo sequences are recommended. For detecting lesions with high inflammatory activity, it is still suggested to employ gadolinium (Gd) enhanced T1w scans ≥5 min after Gd administration. The use of linear gadolinium contrast agents has become somehow scrutinized with reports on persistent Gd deposition in the dentate nucleus and the basal ganglia [21] after repeated application and recently EMA has restricted use of these contrast agents. Whereas clinical relevance is uncertain and also modern macrocyclic Gd agents appear to be more stable, indication for the application of Gd should follow a clear rationale (e.g. clinical symptomatology, new T2-lesions) [7].

Ideally, this imaging approach will yield a quantitative output for the same measures across repeat scans (i.e. lesion counts), and if possible, also volumetric measurements (of T2 lesions as well as brain structures, and whole brain). Here, it may be advised to look for a user-friendly system that automatically generates the requested outputs. One example for for reliable detection and quantification of T2-weighted hyperintense MS lesions is the FLAIRfusion image processing approach. FLAIRfusion provides reliable detection of newly developing MS lesions along with strong inter-reader agreement across all levels of expertise in 35 s of reading time with a combined sensitivity of 100%, and a specificity of 88.2% [48]. In contrast to such automated protocols to quantify inflammatory activity, the assessment of brain atrophy in real-world settings is much more complicated and respective automatic analysis pipelines are under development [45].

Beyond MRI, measures of cognitive information processing (as previously measured by the PASAT-3 test) and quality of life (QoL) measures seemed to be strong prognostic factors as baseline prognostic marker; even stronger than about 20 other baseline variables investigated, including many MRI parameters [42]. This may be due to the fact that cognitive dysfunction rather represents a marker of neurodegeneration than inflammatory activity. According to the concept of brain reserve, neurodegenerative processes in MS may lead to a decrease in intracranial volume with time. Individuals with a larger cognitive reserve may be able to withstand a more severe disease burden without suffering cognitive impairment or dementia [55].

Thus, a cognitive decline on treatment may indicate inadequate disease control. Yet, the absence of cognitive decline may not necessarily mean the reverse (that the disease is adequately controlled) - it may simply indicate that some cognitive reserve is still available and the degenerative processes are hence masked. Unfortunately, this brain reserve may be depleted even during periods of apparent remission if disease activity is not kept under control. In fact, cognitive deterioration is strongly associated with other measures of disease activity, including brain volume change. Recent studies have shown cognition to be a strong and sensitive marker of disease progression over time [35]. It thus is advised to perform cognitive testing on an annual basis [29]. Ideally, all cognitive domains should be assessed with a battery of test. Yet, in clinical practice, this is rarely feasible. If testing only one domain, it is suggested to focus on processing speed. Here, the SDMT is the most commonly recommended test which is increasingly available as a digital tool [1].

In summary, it is important to set a treatment goal and have an agreed threshold for assessing the treatment response together with the individual patient. It is important not to miss activity and to have reliable measures of disease activity as well as neurodegeneration at hand, and to employ them in an optimal way. To date, the best available tool is a standardised quantified MRI which may depict both, inflammatory activity and neurodegeneration. In addition, one may consider the assessment of cognition or QoL to monitor for early deterioration in functional reserve with the ultimate goal not to miss the window of opportunity to optimise immunotherapy before disability accumulates. The combination of different measures as applied in the “no evidence of disease activity” (NEDA) concept or the “multiple sclerosis decision model” that incorporates additional neuropsychological aspects may assist in clinical practice, although not all of those models have been completely validated yet [53].

### Switching, sequencing, and stopping of therapy

Since 2011, three new oral treatment options and three new monoclonal antibodies for the treatment of relapsing remitting MS have been licensed in the EU. This plethora of new therapeutic concepts leads to new questions including issues like sequencing of therapies, how to switch between different compounds, and when to stop a given therapy. Kobelt and co-workers conducted a cross-sectional study in 16 countries which included more than 16.000 participants [27]. Patients reported on their disease, health-related QoL and resource consumption. Adjusted for purchasing power parity, costs were 22.800€ in mild, 37.100€ in moderate and 57.500€ in severe disease. Healthcare accounted for 68, 47%, or 26% of these amounts, respectively. Thus, costs and utility were highly correlated with disease severity, but resource consumption was heavily influenced by healthcare systems organization and availability of services. A single center epoch analysis from Italy revealed that the time from diagnosis to start of treatment was reduced from over 10 years in the 1980s to less than 1 year in the recent years. At the same time, the odds of reaching an EDSS ≥6.0 over the age epoch from 25 to 65 were reduced from over 80% to about 20% - a fact which nicely correlates with the increasing number of available treatment options over time [5].

For sequencing options, different strategies may be envisioned ranking from “slow escalation” with switching between compounds with low or moderate efficacy to “rapid escalation” with immediate switching from a compound with lower or moderate efficacy to a compound with high efficacy. In some cases, a “hit hard an early” strategy with immediate start on a high efficacy compound may be the strategy of choice. In short, there are a large number of possible options, which may crucially depend on disease activity, drug properties and the individual patient profile. When thinking about sequencing or switching strategies, it is important to remember that there are few randomized studies addressing these issues. Many recommendations rely on personal or expert experience only. In fact, the only randomized phase III trial directly addressing a rapid escalation setting was the CARE MS II trial with alemtuzumab treatment in case of interferon beta failure [6]. Yet, due to severe infections and autoimmunity as possible side effects, this very switch may not be the most common choice in every day practice. The mechanism of action of a given compound, information on pharmacokinetics and half-life as well as the potential timeline of reversibility of effects on the immune system are important issues when considering a switch between compounds. Optimally, such strategies are already considered when starting an individual patient on the very first therapy. To get a rough idea, compounds for immune therapy of RRMS may be divided into drugs with anti-proliferative mechanism of action, cell depleting antibodies, therapies interfering with migration or immune cell trafficking and compounds with multiple perceived mechanisms (often grossly called “immunomodulators”). Yet, it is important to remember that even compounds with a perceived “immunomodulatory” mechanism of action may lead to cell depletion as side effect thus bearing the risk of opportunistic infections. Accordingly, some authorities like e.g. the FDA do not follow such classifications. In any case, switching to a new therapy will require an appropriate wash-out with at least normalization of leukocyte or lymphocyte numbers. Yet, a longer wash-out period may bear the inherent risk of returning disease activity especially in higher active patients thus pointing at the importance of pragmatic timelines in individual cases which require thorough information and consenting of patients on risks of their disease versus risks of individual compounds. Finally, it is also important to note that, to date, there are no good routine blood markers to monitor qualitative changes of immune competence. Thus, treating neurologists may have to rely on quantitative numbers of leukocytes or lymphocytes in many situations [25].

Another important issue to consider is the planning of a family and the wish to have children, particularly in higher active patients. This topic comprises conception, pregnancy and lactation [18]. For some compounds like teriflunomide or also fingolimod, some evidence from animal studies point at teratogenic effects. Hence, the summaries of product characteristics recommend strict contraception and advise not to use them during conception or pregnancy. For teriflunomide, an accelerated elimination procedure with an 11 day protocol of cholestyramine or charcoal intake may be applied to yield plasma concentrations below 0.02 mg/L. In case of fingolimod, the wash-put period should comprise at least 2 months. For other compounds including dimethyl fumarate, monoclonal antibodies and injectables, a careful approach weighing the individual risks vs. benefits during pregnancy is recommended. Regarding pregnancy outcomes, prospective datasets for more than 300 pregnancies are only available for the injectables and natalizumab. However, it is worthwhile noting that larger numbers of index cases are necessary to also exclude rarer events. Data on conception under interferon treatment point at a mildly lower birth weight, but no other signs of malformation or miscarriage [56]. In contrast, the experience with natalizumab is more ambiguous and registry data point at an increased overall rate of birth defects, yet with no specific pattern of malformations and no increase in spontaneous abortion rate as compared to the general population [10]. In the case of compounds with a short half-life, like dimethyl fumarate, controlling time periods between stopping treatment and conception seem easier, but still require careful planning. Finally, compounds with pulsed or intermittent application like alemtuzumab or cladribine may offer the chance of pregnancy during drug free intervals after an appropriate safety margin. However, experience on pregnancies after exposure to these compounds is still limited.

During lactation, there are no comprehensive safety data for any compound on the market and patients are mostly advised to rely on immunomodulatory effect of breast feeding or to re-start immunotherapy after ablacation.

After a longer period without relapses, the discontinuation of disease modifying therapy (DMT) is a frequently considered topic in RR-MS. Yet, data on the disease course after stopping treatment are scarce. In a monocentric approach, several factors that could be associated with remaining relapse free after cessation of DMT were analyzed [4]. However, these data warrant further corroboration. In an analysis of the MS Base registry including patients with no relapses for at least 5 years on DMT, 485 patients stopping DMT were compared to 854 patients staying on therapy in a propensity-score matched approach. In this study, the time to first relapse was similar between both groups while the time to confirmed disability progression was significantly shorter among patients stopping DMT than those continuing (adjusted hazard ratio = 1.47, [24]). These data argue for careful evaluation of individual patients and their risk for progression when discussing the discontinuation of DMT. Moreover, the results are well in line with many data from registries pointing at longer term effects of modern immunotherapy on disability progression, particularly in the setting of early treatment initiation in RRMS.

## Conclusion

Patient and disease heterogeneity at the initial presentation and during the disease course renders the increasing treatment choices for RRMS very valuable thus allowing for personalisation of treatment. First, long-term experience on drug safety and efficacy of a compound may inform decision making. Here, real-world data and well-structured registries are of particular importance. Second, optimal monitoring for treatment response is a key issue. Here, it is strongly advised to employ standardized MRI protocols with automated and quantifiable algorithms. In addition, monitoring for cognition, e.g. with the SDMT as screening tool should be considered.

For strategic long-term planning, the timing of reversibility of immune cell effects is of particular interest. In short, the ultimate goal for navigating choices in RRMS management is to choose the right drug for the right patient at the right time.

## Abbreviations

CNS: Central nervous system
DMT: Disease modifying therapy
Gd: Gadolinium
MRI: Magnetic resonance imaging
NAMW: Normal appearing white matter
PIRT: Pulsed immune reconstitution therapy
PML: Progressive multifocal leukoencephalopathy
PPP: Purchasing power parity
PRO: Patient reported outcome
QoL: Quality of life
RRMS: Relapsing-remitting MS
SDMT: Symbol digit modalities test
SIMOA: Single molecule array
